# Rutin, γ-Aminobutyric Acid, Gallic Acid, and Caffeine Negatively Affect the Sweet-Mellow Taste of Congou Black Tea Infusions

**DOI:** 10.3390/molecules24234221

**Published:** 2019-11-20

**Authors:** Jia Li, Yuefeng Yao, Jiaqin Wang, Jinjie Hua, Jinjin Wang, Yanqin Yang, Chunwang Dong, Qinghua Zhou, Yongwen Jiang, Yuliang Deng, Haibo Yuan

**Affiliations:** 1 Key Laboratory of Tea Biology and Resources Utilization, Ministry of Agriculture, Tea Research Institute, Chinese Academy of Agricultural Sciences, Hangzhou 310008, China; jiali1986@tricaas.com (J.L.); yaoyuefeng2018@163.com (Y.Y.); 82101175064@caas.cn (J.W.); huajinjie@tricaas.com (J.H.); jinjinwangtkzc@tricaas.com (J.W.); yangyq@tricaas.com (Y.Y.); dongchunwang@tricaas.com (C.D.); jiangyw@tricaas.com (Y.J.); 2 Graduate School of Chinese Academy of Agricultural Sciences, Beijing 100081, China; 3 Key Laboratory of Microbial Technology for Industrial Pollution Control of Zhejiang Province, College of Environment, Zhejiang University of Technology, Hangzhou 310014, China; qhzhou@zjut.edu.cn

**Keywords:** congou black tea, sweet, mellow, taste flavor, infusion, quantitative analysis, sensory evaluation

## Abstract

The sweet-mellow taste sensation is a unique and typical feature of premium congou black tea infusions. To explore the key taste-active compounds that influence the sweet-mellow taste, a sensory and molecular characterization was performed on thirty-three congou black tea infusions presenting different taste qualities, including the sweet-mellow, mellow-pure, or less-mellow taste. An integrated application of quantitative analysis of 48 taste-active compounds, taste contribution analysis, and further validation by taste supplementation experiments, combined with human sensory evaluation revealed that caffeine, γ-aminobutyric acid, rutin, succinic acid, citric acid, and gallic acid negatively affect the sweet-mellow taste, whereas glucose, sucrose, and ornithine positively contribute to the sweet-mellow taste of congou black tea infusions. Particularly, rutin, γ-aminobutyric acid, gallic acid, and caffeine, which impart the major inhibitory effect to the manifestation of the sweet-mellow taste, were identified as the key influencing components through stepwise screening and validation experiments. A modest level of these compounds was found to be favorable for the development and manifestation of the sweet-mellow taste. These compounds might potentially serve as the regulatory targets for oriented-manufacturing of high-quality sweet-mellow congou black tea.

## 1. Introduction

Tea, processed from fresh leaves of *Camellia sinensis* L., is one of the most popular beverages worldwide attributed to its appealing flavors and beneficial health effects [1,2]. The fermented black tea accounts for up to 78% of tea consumption in the world [3]. Whereas ‘crush, tear, curl’ (CTC) black tea, presented as granular tea leaf particles, is widely consumed in the Western countries, the orthodox Chinese black tea with thin and strip-sharped whole-leaves, called the congou black tea, is traditionally preferred in China. Congou black tea is known for its elaborate and time-consuming manufacturing procedures, including hand-plucking, withering, rolling, fermentation, firing, and further refinement [4]. Infusions of high quality congou black tea exhibit a bright amber-red color, a fragrant aroma, and a sweet-mellow taste [5]. The softly sweet-mellow taste sensation is a unique and typical feature of premium congou black tea infusions, as compared to the strong, full-bodied, and brisk taste rendered by CTC black tea liquor [6]. It can improve the overall palatability and mouthfeel of tea infusions, and thus, is of particular significance to the flavor of the tea, and represents an essential element for high-quality congou black tea [7]. Therefore, a sensory and molecular characterization of the congou black tea infusions and an exploration of the key compounds that influence the manifestation of the sweet-mellow taste sensation would be of interest to expand our knowledge of tea flavors, and provide regulatory clues for the manufacturing of premium congou black tea.

The taste profile perceived by tea tasters, formed by the complex interplay among the basic taste attributes of astringency, bitterness, sourness, sweetness, and umami-like taste, is one of the key criteria in tea quality evaluation. Multiple attempts have been made, which have greatly advanced the understanding of taste flavor and related taste-active compounds of various tea types, including black tea [6,8,9,10,11], green tea [12,13,14,15], oolong tea [16], and Pu-erh tea [17]. Certain non-volatile compounds that impart various taste qualities are possibly responsible for the tea taste profile. The bitter-tasting alkaloid, caffeine, is the key driver to the bitter taste of tea [6,12], whereas the major polyphenols epigallocatechin gallate and flavonol glycosides are the main contributors to tea astringency [6,9,12]. l-glutamate and l-theanine (5-*N*-ethyl glutamine) are related to the umami-like taste of green tea liquor and mat-cha [12,13]. The colored, polymeric theaflavins and thearubigins, generated via condensations of flavan-3-ols during tea fermentation (which is mediated by polyphenol oxidases) are possibly responsible for the astringency and briskness of black tea [18,19,20], albeit with controversial results [8]. However, the sweetness-related taste of tea liquors is less studied. Catechins and gallic acid have been found to be associated with the sweet aftertaste of green tea through human sensory analysis of their aqueous solutions [15,16]. To our best knowledge, by far there is no molecular investigation report that is centered around the sweet-mellow taste of the congou black tea.

In this study, aimed to unravel the key tastants that influence the sweet-mellow taste of congou black tea infusions, a molecular and sensory characterization was performed on thirty-three congou black tea infusions with different taste characteristics (sweet-mellow, mellow-pure, and less-mellow), through an integrated application of quantitative analysis, taste contribution analysis, and further validation by external supplementation experiments of the screened candidate compounds.

## 2. Results and Discussion

### 2.1. Taste Evaluation

Thirty-three congou black tea samples were classified into three groups, including 11, 14, and 8 samples featuring the sweet-mellow (group I), mellow-pure (group II), and less-mellow (group III) taste sensations, respectively (Table 1), according to their descriptive sensory evaluation results obtained by human panelists, with a special focus on the taste attribute sweetness. The sensory evaluation procedure is described in the Materials and Methods section. Tea infusions of group I exhibited a gentle sweet-mellow taste, along with a pleasant fruity or brisk sensation in some infusions. Infusions of group II exhibited a mellow, mild taste, in company with a brisk or thick sensation in some samples, whereas infusions of group III presented an unpleasant less-mellow taste including, ripe, stale, or sour sensations. Significantly higher scores of overall taste quality were rated for the sweet-mellow infusions over the other two groups (Table 1). Detailed information of all enrolled tea samples regarding their taste descriptions, producing areas, and taste scores is provided in the Supplementary Materials (Table S1). The taste difference perceived by human panelists was well in line with that characterized by the taste sensors of electronic tongues in our previous work [7].

### 2.2. Quantitative Analysis and Screening of Candidate Compounds

A total of 48 taste-active compounds (6 flavan-3-ols, 4 theaflavins, 23 free amino acids, 7 flavonol glycosides, 4 organic acid, 2 free sugars, gallic acid, and caffeine) as well as the total soluble sugars were quantified in the studied black tea infusions (Table 2).

**Figure 1:**
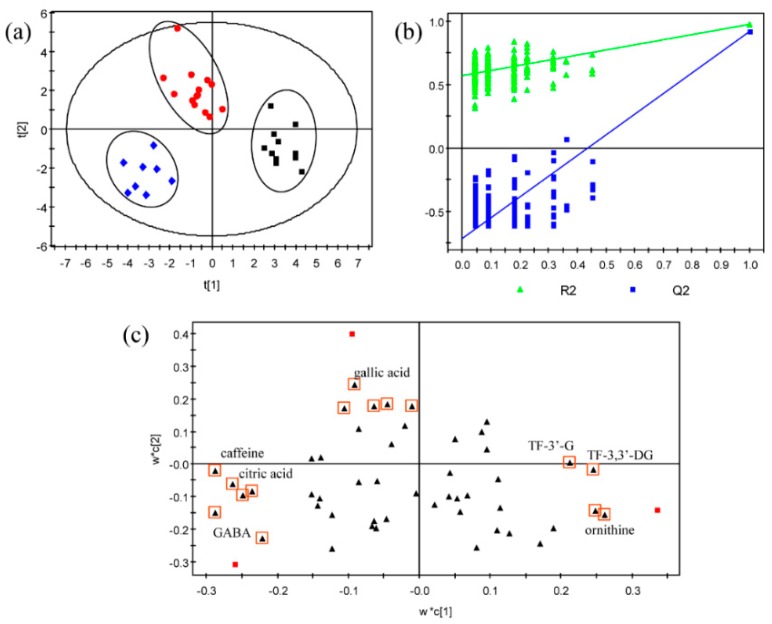
An overview of the chemical composition profiles of black tea infusions featured with the sweet-mellow, mellow-pure, and less-mellow taste quality revealed by partial least square–discriminate analysis (PLS-DA) after orthogonal signal correction (OSC) data filtering. (**a**) Score scatter plot of tea infusion profile composed of quantified taste-active compounds after unit variance (uv) scaling. Black box, red dot, and blue squares represent tea infusions rendering the sweet-mellow, mellow-pure, and less-mellow taste quality, respectively. (**b**) Cross-validation of the PLS–DA model by 200 permutations. (**c**) Loading plot. Black triangles represent all variables and those highlighted with red box indicate compounds that might significantly contribute to group separation. TF-3′-G, theaflavin-3′-gallate; TF-3,3′-DG, theaflavin-3,3′-digallate; GABA,γ-aminobutyric acid.

First, to get a global pattern of the chemical compositions of different black tea infusions, a multivariate analysis of partial least square–discriminate analysis (PLS–DA) was performed after orthogonal signal correction (OSC) data pretreatment. As revealed by the score scatter plot (Figure 1a) of the PLS–DA model (R^2^X = 0.51, R^2^Y = 0.97, Q^2^ = 0.88), an evident clustering of the three groups was achieved. The obtained PLS–DA model was cross-validated by 200 permutations (Figure 1b). This result implied a significant chemical compositional difference among the three groups. To screen the key compounds potentially responsible for the perceived taste difference, a loading scatter plot was generated (Figure 1c), which enabled the visualization of variables highly correlated with the classification patterns [21]. Theaflavin-3′-gallate (TF-3′-G), theaflavin-3,3′-digallate (TF-3,3′-DG), l-ornithine, caffeine, citric acid, γ-aminobutyric acid, and gallic acid might be the key differential compounds.

Furthermore, all quantified tastants were statistically compared (Table 2).

*Flavan-3-ols.* As a major fraction of tea polyphenols, flavan-3-ols, i.e., catechins, provide a unique chemical “signature” of tea as well as the astringent taste [6,22]. Herein, no significant difference was detected of flavan-3-ols for the sweet-mellow infusions against the other tastes (Table 2).

*Theaflavins.* The yellowish-orange theaflavins were considered to contribute to the briskness, astringent taste, and bright color of the black tea liquor [18,20]. Theaflavin and its mono- and di-gallates were quantified, with the digalloyl compound TF-3,3′-DG as the predominant species, accounting for approximately 68% of the total theaflavins in black tea infusions. Significantly higher levels of TF-3′-G and TF-3,3′-DG were detected in sweet-mellow as compared to the less-mellow infusions (1.3 ± 0.5 vs. 0.8 ± 0.4 µmol/L, *p* = 0.019; 16.1 ± 8.4 vs. 9.0 ± 5.1 µmol/L, *p* = 0.016; respectively). Particularly, the concentration of TF-3,3′-DG in sweet-mellow infusions was 1.8 times higher (Table 2).

*Free Amino acids.* Free amino acids are both biologically- and taste-active compounds in tea, which impart the complex taste qualities including umami-like, sweet and bitter tastes [6]. Particularly, l-theanine is the predominant amino acid component that specifically occurs in tea [23]. Herein, twenty-three free amino acids were quantified (Table 2), which were primarily composed of L-theanine, followed by l-asparagine and l-glutamic acid, similar to the composition pattern determined in Chinese black tea in a previous study [24]. Three amino acids varied significantly among the three groups. The content of γ-aminobutyric acid was the lowest in sweet-mellow and was increasingly higher in mellow-pure and less-mellow infusions (17.3 ± 12.4, 26.2 ± 17.8, and 42.9 ± 20.7 µmol/L, *p* = 0.003 and 0.034, respectively). It was notable that l-ornithine showed a significantly higher level in sweet-mellow infusions and was not detectable in either mellow-pure or less-mellow infusions (*p* = 0.005 and 0.013, respectively). Additionally, l-alanine exhibited a significantly lower concentration in sweet-mellow infusions as compared to less-mellow infusions (27.2 ± 12.9 vs. 53.1 ± 16.3 µmol/L, *p* = 0.005).

*Flavonol glycosides.* Flavonol glycosides were identified as the key astringent taste compounds in tea [9]. Seven major flavonol glycosides were quantified. Quercetin-3-*O*-rutinoside, i.e., rutin, exhibited an approximately 1.8 times lower concentration in sweet-mellow infusions as compared to less-mellow infusions (5.7 ± 3.8 vs. 9.9 ± 4.4 µmol/L, *p* = 0.015) (Table 2).

*Soluble Sugars.* Sugars are responsible for the sweetness in food [11]. The major mono- and di- saccharide in tea infusions, glucose, and sucrose, showed a significantly higher level in sweet-mellow infusions in comparison to mellow-pure (263.6 ± 73.4 vs. 197.3 ± 40.3 µmol/L, *p* = 0.002, for glucose) or less-mellow infusions (6.7 ± 9.2 vs. 2.7 ± 0.7 µmol/L, *p* = 0.04, for sucrose) (Table 2). Additionally, the total soluble sugars, comprising mono-, di-, and oligosaccharides, presented a gradually increasing trend from less-mellow towards sweet-mellow taste infusions, however, the trends were not statistically significant (Table 2).

*Organic acids.* Organic acids are the compounds that render the sour taste [6]. Four organic acids were quantified. Among them, succinic acid and citric acid were significantly lower in sweet-mellow infusions against the other tastes. Particularly, the concentration of citric acid was the lowest in sweet-mellow and was successively higher in mellow-pure and less-mellow infusions (122.5 ± 24.4 vs. 143.8 ± 17.3 vs. 168.5 ± 11.0 µmol/L, all *p* < 0.001) (Table 2).

*Others.* The content of alkaloid caffeine was gradually lower from less-mellow towards mellow-pure and sweet-mellow liquors (1581.2 ± 194.9 vs. 1441.8 ± 153.1, 1337.1 ± 79.9 µmol/L, *p* = 0.039 and 0.001, respectively). Similarly, gallic acid showed a significantly lower amount in sweet-mellow compared to mellow-pure infusions (295.0 ± 141.1 vs. 453.8 ± 157.8 µmol/L, *p* = 0.013).

Collectively, twelve differential compounds were screened, which might provide clues to the candidate compounds. They included TF-3′-G, TF-3,3′-DG, L-ornithine, glucose, and sucrose with higher amount in sweet-mellow infusions, as well as L-alanine, γ-aminobutyric acid, rutin, succinic acid, citric acid, gallic acid, and caffeine, which were lower in sweet-mellow infusions. These results to large extent agreed with the results obtained by the PLS–DA analysis (Figure 1c).

However, their concentration differences might have been casual and not essentially associated with different taste perceptions. The comprehensive taste perceived by the gustatory system could be influenced by the combinatorial taste quality and contribution of tastants, as well as the potential interplay amongst them and the complex tea infusion matrix [25]. Thus, an investigation into their taste contribution and further validation of the influence of the screened compounds on the tea taste profile by human bioresponse is needed.

### 2.3. Taste Contribution Analysis

To investigate the taste contribution of individual compounds to the overall tea taste profile, taste activity values, i.e., dose-over-threshold (Dot) factors, were calculated as the ratio of the concentration of each compound in tea infusion to its corresponding taste threshold, either based on previous literature sources [6,13,16,26] or human taste experiments, in case of lack of such data (Table 3). Ten tastants, including TF-3,3′-DG, gallic acid, γ-aminobutyric acid, six flavonol glycosides, and caffeine, which impart puckering or velvety-like astringency, or bitterness, exhibited the major contribution to tea tastes (DOT > 1) (marked with “#” in Table 3). The taste contribution pattern of congou black tea infusions was different from that of unfermented green tea drinks [12] and infusions of semi-fermented oolong tea [16], which might be largely associated with distinct degrees of fermentation. On the other hand, this pattern partially agreed with that of the Darjeeling black tea, as previously reported [6], except for the compounds of TF-3,3′-DG, gallic acid, and γ-aminobutyric acid, which was possibly attributed to different cultivars and the unique manufacturing procedures of the congou black tea.

Among the screened 12 candidate compounds (marked with “*” in Table 3), TF-3,3′-DG, gallic acid, γ-aminobutyric acid, rutin, and caffeine demonstrated Dot factors above 1 (Table 3). Particularly, rutin, which imparts a velvet-like astringency, showed an overwhelmingly high taste activity due to its considerably low taste threshold. Its Dot factor in sweet-mellow infusions (Dot = 4974.2) was significantly lower than that in mellow-pure (Dot = 5668.6) or less-mellow infusions (Dot = 8635.0). γ-aminobutyric acid, which impart astringency, presented a significant taste contribution in mellow-pure (Dot = 1.3) and less-mellow (Dot = 2.1) infusions, while its taste contribution was less pronounced in sweet-mellow tea infusions (Dot = 0.86). TF-3,3′-DG imparts a puckering astringency and rendered a significant taste contribution in sweet-mellow infusions (Dot = 1.2), whereas a relatively minor contribution was observed in the other two groups (Dot = 0.88 and 0.70). Gallic acid and caffeine, which are astringent or bitter-tasting, showed significantly lower taste contributions in sweet-mellow infusions, against the other two taste groups, despite their Dot factors being larger than 1 in all groups.

Other candidate compounds, i.e., sour-tasting succinic acid and citric acid, and sweet-tasting glucose, sucrose, ornithine, and alanine, as well as an astringent compound TF-3′-G, appeared to be of no significant taste contribution (all Dot factors were much lower than 1) (Table 3).

These results suggested that the differential taste contribution imparted by taste-active compounds, mainly astringent and bitter compounds, are possibly associated with the manifestation of the sweet-mellow taste of black tea infusions. However, this result is based on the calculation of Dot factors using taste thresholds, which is evaluated by using an aqueous solution of each single compound. Since potential interplay, for instance, synergistic effects, might take place among tastants, a further validation of these candidate compounds in a complex matrix of black tea infusions is still needed.

### 2.4. Validation by Taste Supplementation Experiments

To explore the influence of candidate compounds on the sweet-mellow taste of black tea infusions, taste supplementation experiments in combination with human sensory trials were performed, based on the screening results. Aqueous solutions of individual candidate compounds at a series of concentrations were added into the background black tea infusions, to evaluate the taste transformations induced by the supplementation of selected compounds. Sweet-mellow or mellow-pure black tea infusions with a minimal or relative low content of the tested compounds were employed as background tea infusion. The required supplementation concentration (calculated as the supplementation amount/volume of tea infusion) was recorded when a significant taste transformation could be just detected by at least 70% of the assessors. The detailed experimental procedure is described in the Materials and Methods section.

As shown in Table 4, the human bio-response analysis revealed diverse taste changes of background tea infusions upon external supplementation of candidate compounds. The supplementation of bitter-tasting caffeine at a concentration of 515 µmol/L gave rise to a detectable taste transformation of the sweet-mellow background tea infusion, presenting suppressed sweetness, and increased bitterness. Two differential theaflavins, TF-3′-G and TF-3,3′-DG, significantly altered the taste of mellow-pure background infusions by enhancing the astringency when the supplementation concentration was increased to 7.0 and 12.6 µmol/L, respectively. The taste transformation of sweet-mellow background tea infusions was also detected upon the supplementation of γ-aminobutyric acid and rutin, at a concentration of 19.0 and 0.0023 µmol/L, respectively, showing a reduced sweetness and increased astringency and bitterness. Similarly, sour compounds succinic acid and citric acid significantly diminished the sweet taste while giving rise to a detectable sour taste in the background of the sweet-mellow tea infusion, at a supplementation concentration of 450 and 325 µmol/L, respectively. Additionally, gallic acid also showed a negative effect on the sweet-mellow taste by weakening its sweet taste while increasing the sour, astringent taste at a supplementation concentration of 200 µmol/L. As the supplementation concentration of these compounds went further up, further taste transformations occurred with a drastic reduction of the sweet taste and an enhancement of the bitterness, astringency, and sour taste, towards unpleasant “off-flavor”. On the other hand, glucose, sucrose, alanine, and ornithine induced a taste transformation of mellow-pure background infusions, with a detectable increase of the sweet taste when a concentration of 45,000.0, 24,000.0, 23,546.0, and 11,346.0 µmol/L was added, respectively. Further enhancement of the sweet taste of background tea infusions was perceived upon the continuous increase of their supplementation concentrations. No describable taste difference was detected when lower concentrations were applied for all tested compounds. Among all tested compounds, 9 tastants demonstrated influences on the taste profile of black tea infusions, which well-conformed to their quantitative differences of sweet-mellow, in comparison to mellow-pure or less-mellow black tea infusions (Table 4). However, TF-3′-G, TF-3,3′-DG, and alanine triggered taste transformations that were somehow contradictory to their quantitative differences, i.e., higher levels of TF-3′-G and TF-3,3′-DG, as well as lower levels of alanine were presented in the sweet-mellow tea infusions. Thus, the quantitative analysis combined with validation by taste supplementation experiments revealed that caffeine, γ-aminobutyric acid, rutin, succinic acid, citric acid, and gallic acid were negatively correlated to the sweet-mellow taste, whereas glucose, sucrose, and ornithine were positively correlated to the sweet-mellow taste of tea infusions.

In addition, huge variations, spanning several orders of magnitude in supplementation concentrations were observed among the 12 candidates for a detectable taste transformation (Table 4). The supplementation concentration of rutin, γ-aminobutyric acid, gallic acid, and caffeine for a taste transformation from a sweet-mellow taste towards a decreased sweetness, as well as an increased bitterness or astringency were 0.0023, 19.0, 200.0, and 515.0 µmol/L, respectively, corresponding to the final concentrations, which remained within the reasonable range of their “natural” concentrations (Table 4). These results indicated that the sweet-mellow taste might be more susceptible to content changes of rutin, γ-aminobutyric acid, gallic acid, and caffeine.

In contrast, despite the positive effects of glucose, sucrose, and ornithine on the sweet-mellow taste, a considerably higher amount over their “natural” concentrations in tea infusions (approximately 120–25,000 folds higher) (Table 4) was needed to induce a significant improvement in sweetness. These results suggested that the native approaches, for instance, selection of tea cultivars or optimization of primary processing methods, could hardly enrich the content of these compounds and consequently improve the sweet-mellow taste of the congou black tea. Instead, it could be expected that an artificial supplementation at high concentrations would be needed for this purpose.

**Figure 2:**
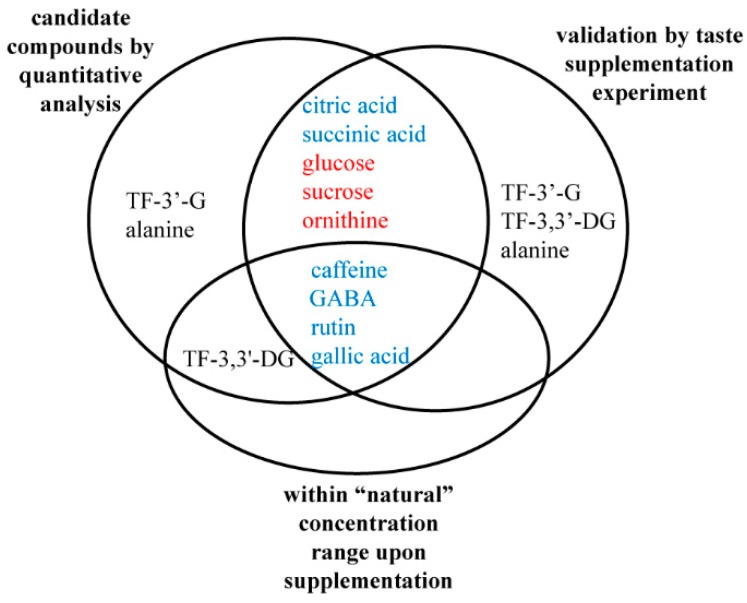
Venn diagram showing the stepwise screening and validation experiments of the key compounds affecting the sweet-mellow taste of the congou black tea infusions. The compounds marked in blue and red represent the compounds that were negatively and positively correlated with the sweet-mellow taste of congou black tea infusions, respectively. TF-3′-G, theaflavin-3′-gallate; TF-3,3′-DG, theaflavin-3,3′-digallate; GABA,γ-aminobutyric acid.

Collectively, the four key components affecting the sweet-mellow taste of congou black tea infusions, i.e., rutin, γ-aminobutyric acid, gallic acid, and caffeine, were identified by stepwise screening and validation experiments (Figure 2). Supplementation of these compounds at even relatively low levels, corresponding to the final concentrations that remain within the reasonable range of their “natural” concentrations, could induce detrimental transformations of the sweet-mellow taste. These four compounds were, to some extent, overlapped with those compounds presenting major taste contributions (Dot > 1). Among these, rutin, γ-aminobutyric acid, and gallic acid imparted puckering or a velvet-like astringency, while caffeine presented a bitter taste. It is interesting to notice that three of them shared the taste quality of astringent sensation, implying the significant negative effect of astringency on the sweet-mellow taste. It agreed with a previous study reporting that astringency showed a greater inhibitory effect on the development of the sweet aftertaste in green tea infusions, as compared to other taste qualities [14]. A recent study showed that gallic acid contributed to the sweet aftertaste [15], however, its required concentration was much higher than the “natural” concentration detected in black tea infusions in our study. Thus, despite multiple taste-active roles, gallic acid demonstrated a more prominent suppressive effect on the sweet-mellow taste rather than a positive effect.

It could be inferred from these results that, a modest level of these four key affecting components was favorable for the development and manifestation of sweet-mellow taste, and might present as a prerequisite for the sweet-mellow taste. It was reported that the different tea cultivars showed distinct enrichment patterns of these compounds [27], and their levels were decreased during the congou black tea manufacturing process [28]. Hence, these four compounds presenting the major inhibitory effects might serve as the potential regulatory targets for manufacturing sweet-mellow congou black tea by employing appropriate tea cultivars and optimizing the processing methods/parameters.

## 3. Materials and Methods

### 3.1. Chemicals and Reagents

Liquid chromatography grade solvents and reagents of acetonitrile, formic acid, and acetic acid were purchased from TEDIA (Fairfield, OH, USA), or Tokyo Chemical Industry (Chuo-ku, Japan). Authentic standards of all analyzed compounds were purchased from Jinsui biotechnology (Shanghai, China), Yuanye bio-technology (Shanghai, China), J&K scientific (Beijing, China), ZZBIO (Shanghai, China), or Sigma-Aldrich (St Louis, MO, USA). Detailed information of the CAS number, supplier and purity of the standards is provided in the Supplementary Materials (Table S2).

### 3.2. Collection of Congou Black Tea Samples

The congou black tea samples used in this study were selected from our in-house tea bank based on their taste characteristics, as evaluated by the human panelists (described in Section 3.4.1). A total of 33 congou black tea samples were enrolled, which were collected from the Hubei province (Yichang, 110°15′–112°04′ E, 29°56′–31°34′ N), the Anhui province (Qimen, 117°12′–117°57′ E, 29°35′–30°08′ N), the Zhejiang province (Hangzhou, 118°21′–120°30′ E, 29°11′–30°33′ N; Lishui, 118°41′–120°26′ E, 27°25′–28°57′ N), the Yunnan province (Lincang, 98°40′–100°32′ E, 23°05′–25°03′ N), and the Sichuan province (Yibin, 103°36′–105°20′ E, 27°50′–29°16′ N), China.

### 3.3. Preparation of Tea Infusions

Tea infusions were prepared by brewing 3.0 g of black tea in a 150 mL cup specialized for tea tasting, using pre-heated pure water at 85 °C for 4 min, which were then transferred to a clean bowl. Tea infusions were subjected to sensory evaluation after cooling down to about 50 °C. For the composition analysis, tea infusions were cooled down to room temperature and filtered through 0.2 µm Millipore membrane filters before injection.

### 3.4. Human Sensory Evaluation

#### 3.4.1. Tea Sensory Evaluation

All tea infusions were evaluated and scored in the sensory attributes of taste, aroma, and color, by a group of panelists composed of three male and two female certificated experts from the Tea Research Institute of Chinese Academy of Agricultural Sciences, according to the “National Methodology of Sensory Evaluation of Tea” of China [29]. The evaluations were performed in a clean, bright and quiet tasting room, which was 15 m^2^ large and was kept at 22 °C. All congou black samples were served in a random order. None of the panelists had prior knowledge of these tested tea samples. Detailed technical aspects of the sensory analysis are described in the Supplementary Materials.

#### 3.4.2. Taste Supplementation Experiments

The taste transformation of background black tea infusions upon the supplementation of the candidate compounds were evaluated by 6–9 trained assessors using a triangle test, as described previously [9]. None of the assessors had any prior knowledge of the identity, or taste quality, or supplementation concentration of the tested compounds. Sweet-mellow or mellow-pure black tea infusions with a minimal or relative low content of the tested compounds were employed as the background tea infusion for taste transformation evaluation. For each round of triangle test, one black tea infusion with supplementation and two without supplementation were presented to the assessors. A series of supplementation concentrations were performed and served in random order. One teaspoon of tested tea infusions (about 5 mL) was applied, swirled in the mouth then spitted out. The assessors were then asked to distinguish and describe the taste difference. The supplementation concentration at which a taste transformation could be just correctly detected by more than 70% of the assessors was recorded. To prevent fatigue and potential carry-over interferences, each assessor rinsed the mouth using pure water during the sample-to-sample interval.

### 3.5. Analysis of Chemical Compositions

#### 3.5.1. Catechins, Gallic Acid, Caffeine, and Theaflavins

The measurement of catechins, gallic acid, caffeine, and theaflavins was performed on an HPLC system (LC-20A, Shimadzu, Kyoto, Japan) equipped with an ultraviolet detector. Separation was carried out on a symmetry C18 column (4.6 × 250 mm, 5 µm, Waters) using solvent A (2% acetic acid in water) and solvent B (100% acetonitrile), at column temperatures of 35 °C. For analysis of catechins, gallic acid, and caffeine, solvent B was linearly increased from 6.5 to 15% from 0 to 16 min, then linearly increased to 25% from 16 to 25 min, followed by equilibration at 6.5% for 5 min for the next injection, at a flow rate of 1 mL/min. The detection wavelength was set at 280 nm. For analysis of theaflavins, solvent B was linearly increased from 20 to 25% during 35 min and maintained for 3 min, and then equilibrated at 20% for 3 min for the next injection, at flow rate of 1.5 mL/min. The detection wavelength was set at 380 nm. They were quantified using calibration curves obtained from authentic standards.

#### 3.5.2. Flavonol Glycosides

The measurement of flavonol glycosides was performed on an HPLC system (1100, Agilent) using a symmetry C18 column (4.6 × 250 mm, 5 µm, Waters). The LC separation was started with 6% solvent B (100% acetonitrile) and 94% solvent A (0.15% formic acid in water), at flow rate of 1 mL/min. Solvent B was linearly increased to 17% of B from 0 to 2 min, and further increased to 19% from 2 to 22 min, followed by an increase to 30% in 1 min, which was maintained for 2 min, and then equilibrated at 6% for 5 min, for the next injection. The detection wavelength was set at 360 nm. All detected flavonol glycosides were quantified using the calibration curves obtained from authentic standards.

#### 3.5.3. Free Amino Acids

The free amino acids were determined by high-performance cation-exchange chromatography with postcolumn derivatization, using an automatic amino acid analyzer (S-433D, Sykam, Germany) equipped with a cation separation LCAK07/Li column (4.6 mm × 150 mm, Sykam), as previously described with modifications [30]. Details of the chromatography is provided in the Supplementary Materials.

#### 3.5.4. Free Sugars and Organic Acids

The measurement of glucose, sucrose, succinic acid, oxalic acid, malic acid, and citric acid was performed on an ultra-high-performance liquid chromatography (UHPLC) coupled to a quadrupole Orbitrap mass spectrometry system (Q Exactive, Thermo, Carlsbad, CA, USA). The LC separation was performed on an ACQUITY UPLC HSS T3 column (2.1 × 100 mm, 1.8 µm, Waters), as previously described [27]. The gradient separation was started with 5% solvent B (acetonitrile with 0.1% formic acid) and 95% solvent A (water with 0.1% formic acid) and was maintained for 2 min. Next, it was linearly increased to 12% B in 1 min and further to 25% B in next 7 min, and finally to 98% B in 2 min, which was maintained for 1 min, followed by equilibration at 5% B for 2 min for the next injection. The column temperature was 35 °C and the flow rate was 0.35 mL/min. These compounds were identified using accurate mass, MS/MS fragmentation, and authentic standards confirmation. All compounds were quantified using the calibration curves obtained from authentic standards.

The total content of soluble sugars was determined by the anthrone–sulfuric method [31].

### 3.6. Data Processing and Statistics

The multivariate analysis partial least square–discriminate analysis (PLS–DA) was performed using SIMCA-P 11.5 (Umetrics AB, Umea, Sweden), after spectral filtering by orthogonal signal correction (OSC) [21] and unit variance scaling. Statistical significance was determined by one-way ANOVA with LSD post-hoc test using SPSS 18.0 (IBM, Armonk, NY, USA). *p* < 0.05 was set as the statistical significance level.

## 4. Conclusions

To summarize, an integrated application of quantitative analysis, taste contribution analysis, and validation by taste supplementation experiments, combined with human sensory evaluation revealed that caffeine, γ-aminobutyric acid, rutin, succinic acid, citric acid, and gallic acid negatively affect the sweet-mellow taste, whereas glucose, sucrose, and ornithine positively contribute to the sweet-mellow taste of congou black tea infusions. Particularly, rutin, γ-aminobutyric acid, gallic acid, and caffeine, which impart the major inhibitory effect to the manifestation of sweet-mellow taste, were identified as the key influencing components. Their content variations which remained in the reasonable range of their “natural” concentrations could induce significant taste transformations of sweet-mellow infusions. This study was expected to open new insights into tea taste flavor and to provide regulatory targets for manufacturing of high-quality, sweet-mellow congou black tea.

## Figures and Tables

**Table 1:** Characteristics of the thirty-three congou black tea samples enrolled in this study, including their production areas, taste characteristics, and the overall taste scores evaluated by human panelists.

Group	Taste Description	Sample Number	Producing Area	Taste Score
Group I: sweet-mellow	sweet, mellow, some with a fruity or brisk sensation	11	Zhejiang (5), Hubei (3), Yunnan (2), Anhui (1)	88.1 ± 2.9^a^
Group II: mellow-pure	mellow, mild, some with a brisk or thick sensation	14	Hubei (9), Zhejiang (2), Sichuan (2), Anhui (1)	84.6 ± 3.1^b^
Group III: less-mellow	with unpleasant tastes including ripe, stale, or sour sensation	8	Hubei (3), Anhui (2), Yunnan (2), Sichuan (1)	82.3 ± 2.7^b^

^a, b,^ Different letters indicate significant differences between mean values (*p* < 0.05). Statistical significance was determined by one-way ANOVA with LSD post-hoc test.

**Table 2:** Quantitative analysis and comparison of taste-active compounds among the sweet-mellow (I), mellow-pure (II), and less-mellow (III) black tea infusions. Data are presented as mean ± SD (*n* = 11, 14, 8, respectively). Statistical significance was determined by one-way ANOVA through an LSD post-hoc test. n.s., not significant.

Components in Tea Infusion	Sweet-Mellow (I)	Mellow-Pure (II)	Less-Mellow (III)	*p Values*		
	Mean ± SD (µmol/L)	Mean ± SD (µmol/L)	Mean ± SD (µmol/L)	I vs. II	I vs. III	II vs. III
Flavan-3-ols						
epigallocatechin, EGC	0.0 ± 0.0	4.2 ± 9.2	0.0 ± 0.0	n.s.	n.s.	n.s.
catechin, C	2.8 ± 9.3	2.9 ± 5.0	0.0 ± 0.0	n.s.	n.s.	n.s.
epigallocatechin gallate, EGCG	7.1 ± 7.8	17.9 ± 39.6	5.6 ± 8.8	n.s.	n.s.	n.s.
epicatechin, EC	84.8 ± 53.6	83.8 ± 54.1	52 ± 16.4	n.s.	n.s.	n.s.
gallocatechin gallate, GCG	5.9 ± 4.6	4.9 ± 2.5	8.6 ± 4.3	n.s.	n.s.	0.037
epicatechin gallate, ECG	72.5 ± 48.4	107.0 ± 79.3	58.4 ± 43.0	n.s.	n.s.	n.s.
Theaflavins						
theaflavin, TF	2.0 ± 0.7	1.8 ± 0.8	1.5 ± 1.0	n.s.	n.s.	n.s.
theaflavin-3-gallate, TF-3-G	4.8 ± 2.1	3.5 ± 1.1	4.0 ± 2.8	n.s.	n.s.	n.s.
theaflavin-3′-gallate, TF-3′-G	1.3 ± 0.5	1.0 ± 0.4	0.8 ± 0.4	n.s.	0.019	n.s.
theaflavin-3,3′-digallate, TF-3,3′-DG	16.1 ± 8.4	11.4 ± 3.6	9.0 ± 5.1	n.s.	0.016	n.s.
Amino acids						
phospho-l-serine	11.8 ± 3.1	12.5 ± 4.3	14.7 ± 4.5	n.s.	n.s.	n.s.
l-aspartic acid	40.8 ± 16.1	47.6 ± 19.8	50.5 ± 13.2	n.s.	n.s.	n.s.
l-threonine	16.7 ± 8.9	17.8 ± 11.1	24.1 ± 8.1	n.s.	n.s.	n.s.
l-serine	51.9 ± 31.2	49.5 ± 30.2	65.0 ± 16.8	n.s.	n.s.	n.s.
l-asparagine	286.2 ± 223.2	171.9 ± 168.3	206.2 ± 145.1	n.s.	n.s.	n.s.
l-glutamic acid	97.6 ± 52.4	90.1 ± 58.3	120.1 ± 37.7	n.s.	n.s.	n.s.
l-theanine	339.6 ± 154.3	345.8 ± 167.7	442.3 ± 182.3	n.s.	n.s.	n.s.
l-proline	26.0 ± 17.1	24.6 ± 16.0	24.7 ± 8.7	n.s.	n.s.	n.s.
l-glycine	3.7 ± 3.3	2.4 ± 3.4	3.2 ± 3.8	n.s.	n.s.	n.s.
l-alanine	27.2 ± 12.9	42.0 ± 22.1	53.1 ± 16.3	n.s.	0.005	n.s.
α-aminobutyric acid	1.8 ± 3.7	3.1 ± 5.0	6.0 ± 6.9	n.s.	n.s.	n.s.
l-valine	35.9 ± 20.1	32.0 ± 18.3	35.1 ± 13.9	n.s.	n.s.	n.s.
l-cystine	18.6 ± 10.7	17.0 ± 9.7	18.8 ± 8.9	n.s.	n.s.	n.s.
l-methionine	19.9 ± 11.4	23.3 ± 13.1	28.0 ± 11.7	n.s.	n.s.	n.s.
l-isoleucine	3.3 ± 6.4	9.2 ± 9.8	6.7 ± 10.9	n.s.	n.s.	n.s.
l-leucine	7.9 ± 7.3	5.5 ± 8.0	7.3 ± 6.7	n.s.	n.s.	n.s.
l-tyrosine	35.4 ± 18.1	27.7 ± 14.2	32.0 ± 11.6	n.s.	n.s.	n.s.
γ-aminobutyric acid, GABA	17.3 ± 12.4	26.2 ± 17.8	42.9 ± 20.7	n.s.	0.003	0.034
l-histidine	3.9 ± 2.4	2.8 ± 2.3	3.3 ± 1.3	n.s.	n.s.	n.s.
l-tryptophan	16.7 ± 9.2	11.5 ± 6.4	11.6 ± 5.2	n.s.	n.s.	n.s.
l-ornithine	1.1 ± 1.6	0.0 ± 0.0	0.0 ± 0.0	0.005	0.013	n.s.
l-lysine	17.7 ± 12.1	14.5 ± 9.3	17.3 ± 8.2	n.s.	n.s.	n.s.
l-arginine	19.2 ± 12.3	15.8 ± 12.5	27.1 ± 25.4	n.s.	n.s.	n.s.
Flavonol glycosides						
vitexin-4”-*O*-glucoside	1.7 ± 0.6	1.8 ± 0.6	2.0 ± 0.7	n.s.	n.s.	n.s.
myricetin 3-*O*-galactoside	4.8 ± 2.8	5.9 ± 3.9	5.4 ± 4.7	n.s.	n.s.	n.s.
vitexin-2-*O*-rhamnoside	2.4 ± 1.5	2.1 ± 0.8	2.7 ± 2.3	n.s.	n.s.	n.s.
quercetin-3-*O*-rutinoside, rutin	5.7 ± 3.8	6.5 ± 2.7	9.9 ± 4.4	n.s.	0.015	0.037
quercetin-3-*O*-glucoside	6.7 ± 2.3	9.0 ± 3.5	10.1 ± 4.8	n.s.	n.s.	n.s.
kaempferol-3-*O*-rutinoside	5.1 ± 3.9	7.3 ± 4.5	5.4 ± 2.7	n.s.	n.s.	n.s.
kaempferol-3-*O*-glucoside	3.7 ± 3.4	6.4 ± 4.7	4.9 ± 2.9	n.s.	n.s.	n.s.
Organic acid						
succinic acid	21.1 ± 6.8	22.2 ± 3.6	24.9 ± 3.5	n.s.	0.02	n.s.
oxalic acid	629.9 ± 147.9	593.6 ± 82.5	600.0 ± 60.0	n.s.	n.s.	n.s.
malic acid	104.1 ± 29.9	99.2 ± 12.8	116.9 ± 12.2	n.s.	n.s.	0.026
citric acid	122.5 ± 24.4	143.8 ± 17.3	168.5 ± 11.0	0.001	<0.001	<0.001
Soluble sugars						
glucose	263.6 ± 73.4	197.3 ± 40.3	231.2 ± 64.6	0.002	n.s.	n.s.
sucrose	6.7 ± 9.2	3.2 ± 2.1	2.7 ± 0.7	n.s.	0.04	n.s.
total of soluble sugars*	879.5 ± 626.2	845.7 ± 605.0	573.3 ± 111.3	n.s.	n.s.	n.s.
Others						
gallic acid	295.0 ± 141.1	453.8 ± 157.8	326.3 ± 146.9	0.013	n.s.	n.s.
caffeine	1337.1 ± 79.9	1441.8 ± 153.1	1581.2 ± 194.9	n.s.	0.001	0.039

*, the concentration unit for the total content of soluble sugars is µg/mL.

**Table 3:** Taste contribution of tea components in the sweet-mellow (I), mellow-pure (II), and less-mellow (III) black tea infusions, evaluated by dose-over-threshold (Dot) factors, which are calculated as the ratio of the concentration of each compound in tea infusion to its corresponding taste threshold. Data presented is mean value of Dot factors (*n* = 11, 14, and 8, respectively).

Tastant	Dot Values			Threshold
	Sweet-Mellow (I)	Mellow-Pure (II)	Less-Mellow (III)	(µmol/L)
**Group 1: Compounds Imparting Puckering Astringency and Rough Oral Sensation**				
epigallocatechin, EGC	0.000	0.008	0.000	520a
catechin, C	0.007	0.007	0.000	410a
epigallocatechin gallate, EGCG	0.037	0.094	0.029	190a
epicatechin, EC	0.091	0.090	0.056	930a
gallocatechin gallate, GCG	0.015	0.013	0.022	390a
epicatechin gallate, ECG	0.279	0.412	0.225	260a
theaflavin, TF	0.125	0.113	0.093	16a
theaflavin-3-gallate, TF-3-G	0.322	0.234	0.266	15a
theaflavin-3′-gallate, TF-3′-G*	0.087	0.068	0.055	15a
theaflavin-3,3′-digallate, TF-3,3′-DG*#	1.236	0.877	0.692	13a
gallic acid*#	1.010	1.554	1.117	292b
**Group 2: Compounds Imparting Mouth-Drying and Velvet-like Astringency**				
γ-aminobutyric acid*#	0.864	1.311	2.146	20a
myricetin 3-*O*-galactoside#	1.787	2.197	1.982	2.7a
vitexin-2-*O*-rhamnoside	0.871	0.765	0.949	2.8a
quercetin-3-*O*-rutinoside, rutin*#	4974.214	5668.570	8635.034	0.00115a
quercetin-3-*O*-glucoside #	10.276	13.913	15.563	0.65a
kaempferol-3-*O*-rutinoside #	20.234	29.248	21.743	0.25a
vitexin-4″-*O*-glucoside #	8.917	8.917	10.701	0.11e
kaempferol-3-*O*-glucoside #	5.460	9.534	7.338	0.67a
theanine	0.057	0.058	0.074	6000a
**Group 3: Bitter-tasting compounds**				
caffeine*#	2.674	2.884	3.162	500a
valine	0.002	0.002	0.002	21,000a
isoleucine	0.000	0.001	0.001	11,000a
leucine	0.001	0.000	0.001	12,000a
tyrosine	0.007	0.006	0.006	5000a
histidine	0.000	0.000	0.000	45,000b
tryptophan	0.004	0.003	0.003	4400c
lysine	0.000	0.000	0.000	80,000b
arginine	0.000	0.000	0.000	75,000b
epigallocatechin gallate, EGCG	0.019	0.047	0.015	380a
epicatechin, EC	0.091	0.090	0.056	930a
gallocatechin gallate, GCG	0.015	0.013	0.022	390a
**Group 4: Umami-like taste compounds**				
glutamic acid	0.033	0.030	0.040	3000a
aspartic acid	0.010	0.012	0.013	4000a
theanine	0.014	0.014	0.018	24,000d
asparagine	0.006	0.003	0.004	50,000b
**Group 5: Sweet-like taste compounds**				
glucose*	0.003	0.002	0.003	90,000a
sucrose*	0.000	0.000	0.000	24,000a
threonine	0.000	0.000	0.001	40,000a
serine	0.002	0.002	0.002	30,000a
proline	0.001	0.001	0.001	26,000a
glycine	0.000	0.000	0.000	30,000a
alanine*	0.003	0.005	0.007	8000a
methionine	0.004	0.005	0.006	5000a
ornithine*	0.000	0.000	0.000	3500a
theanine	0.014	0.014	0.018	24,000d
**Group 6: Sour taste compounds**				
succinic acid*	0.023	0.025	0.028	900a
oxalic acid	0.112	0.106	0.107	5600a
malic acid	0.028	0.027	0.032	3700a
citric acid*	0.047	0.055	0.065	2600a

a, taste thresholds from: Scharbert, S.; Hofmann, T., Molecular definition of black tea taste by means of quantitative studies, taste reconstitution, and omission experiments. *J. Agric. Food Chem.* **2005,** 53, 5377–5384. b, taste thresholds from: Hufnagel, J.C.; Hofmann, T., Quantitative reconstruction of the nonvolatile sensometabolome of a red wine. *J. Agric. Food Chem.* **2008,** 56, 9190–9199. c, taste thresholds from: Liu, P.-P.; Yin, J.-F.; Chen, G.-S.; Wang, F.; Xu, Y.-Q., Flavor characteristics and chemical compositions of oolong tea processed using different semi-fermentation times. *Journal of Food Science and Technology-Mysore* **2018,** 55, 1185–1195. d, taste thresholds from: Kaneko, S.; Kumazawa, K.; Masuda, H.; Henze, A.; Hofmann, T., Molecular and sensory studies on the umami taste of Japanese green tea. *J. Agric. Food Chem.* **2006,** 54, 2688–2694. e, taste threshold evaluated by human panelists in our lab. *, candidate compounds which were significantly differential among the three groups of tea infusions obtained by quantitative analysis. #, compounds with great taste contribution to tea taste (Dot > 1).

**Table 4:** Influence of 12 individual candidate compounds on the taste profile of sweet-mellow or mellow-pure background black tea infusions, revealed by supplementation experiments and human sensory evaluation. Supplementation concentration for taste transformation indicates the lowest supplementation concentration required for a detectable taste transformation by more than 70% of the assessors, which is calculated as the supplementation amount/volume of tea infusion. “Natural” concentration indicated the concentration range detected in the congou black tea infusions in this study.

Compound Supplemented	Taste Quality of Background Tea Infusion	Concentration of the Tested Compound in Background Tea Infusion (µmol/L)	Supplementation concentration for Taste Transformation (µmol/L)	Recognition Rate	Taste Transformation after Supplementation	Whether Consistent in Quantitative Difference	“Natural” Concentration (µmol/L)	Whether Final Concentration Is within the Range of “Natural” Concentration
caffeine	sweet-mellow	1220.8	515.0	100%	sweetness-, bitterness+	Yes	1152–1861.7	Yes
TF-3′-G	mellow-pure	0.7	7.0	71%	sweetness-, astringency+	No	0.3–1.9	No
TF-3,3′-DG	mellow-pure	6.0	12.6	83%	sweetness-, astringency+	No	2.6–27.9	Yes
γ-aminobutyric acid, GABA	sweet-mellow	18.8	19.0	80%	sweetness-, astringency+, bitterness+	Yes	2.6–70.1	Yes
quercetin-3-*O*-rutinoside, rutin	sweet-mellow	1.3	0.0023	100%	sweetness-, astringency+, bitterness+	Yes	1.3–16.9	Yes
succinic acid	sweet-mellow	19.6	450.0	100%	sweetness-, sour+	Yes	14.1–38.8	No
citric acid	sweet-mellow	119.5	325.0	86%	sweetness-, sour+	Yes	89.5–186.7	No
gallic acid	sweet-mellow	93.7	200.0	71%	sweetness-, sour+, astringency+	Yes	93.7–830.9	Yes
glucose	mellow-pure	144.9	45,000.0	86%	sweetness+	Yes	82.0–372.8	No
sucrose	mellow-pure	1.8	24,000.0	100%	sweetness+	Yes	0.93–34.7	No
alanine	mellow-pure	11.6	23,546.0	78%	sweetness+	No	8.5–75.1	No
ornithine	mellow-pure	n.d.	11,346.0	100%	sweetness+	Yes	0–3.9	No

## Supplementary Materials

The following is available online. Table S1: Detailed information of all enrolled 33 tea samples regarding their taste descriptions, production areas, and taste scores. Table S2: Detailed information of the CAS number, supplier, and purity of all standards used in this study. Detailed technical aspects of tea sensory evaluation. Details on the analysis of free amino acids.
