# Supplementary material for: Rutin, γ-Aminobutyric Acid, Gallic Acid, and Caffeine Negatively Affect the Sweet-Mellow Taste of Congou Black Tea Infusions
Source: Molecules. 2019 Nov 20;24(23):4221. doi: 10.3390/molecules24234221 (PMC6930661; doi:10.3390/molecules24234221)
Supplement: Supplementary file 1 [file molecules-24-04221-s001.pdf]

Supplementary materials for publication

Rutin, γ-Aminobutyric Acid, Gallic acid and Caffeine Negatively Affect the Sweet-Mellow Taste of Congou Black Tea Infusions

Jia Li[a], Yuefeng Yao[a,b], Jiaqin Wang[a,b], Jinjie Hua[a], Jinjin Wang[a], Yanqin Yang[a], Chunwang Dong[a], Qinghua Zhou[c], Yongwen Jiang[a], Yuliang Deng[a,*], Haibo Yuan[a,*]

[a] Key Laboratory of Tea Biology and Resources Utilization, Ministry of Agriculture, Tea Research Institute, Chinese Academy of Agricultural Sciences, Hangzhou 310008 China

[b] Graduate School of Chinese Academy of Agricultural Sciences, Beijing 100081 China

[c] Key Laboratory of Microbial Technology for Industrial Pollution Control of Zhejiang Province, College of Environment, Zhejiang University of Technology, Hangzhou 310014 China

* Correspondence should be addressed to:

* Yuliang Deng, Email: donny@tricaas.com

* Prof. Haibo Yuan, Email: 192168092@tricaas.com

Table S1 Detailed information of enrolled 33 congou black tea samples including their producing area, taste descriptions of corresponding tea infusions evaluated by human panelist, and grouping information.

Nr.	Producing Area	Taste Description	Group
1	Hangzhou, Zhejiang	mellow, sweet, brisk	group I
2	Hangzhou, Zhejiang	mellow, sweet	group I
3	Hangzhou, Zhejiang	mellow, sweet, brisk	group I
4	Hangzhou, Zhejiang	mellow, sweet	group I
5	Yichang, Hubei	ripe, stale, a bit sour	group III
6	Yichang, Hubei	ripe, stale	group III
7	Hangzhou, Zhejiang	mellow, pure, mild,	group II
8	Lincang, Yunnan	mellow, sweet	group I
9	Qimen, Anhui	ripe, stuffy	group III
10	Qimen, Anhui	mellow, mild	group II
11	Qimen, Anhui	a bit sour	group III
12	Qimen, Anhui	mellow, sweet	group I
13	Enshi, Hubei	mellow, pure, mild	group II
14	Lishui, Zhejiang	mellow, sweet, fruity	group I
15	Lishui, Zhejiang	mellow, thick	group II
16	Yibin, Sichuan	mellow, brisk	group II
17	Yibin, Sichuan	sour	group III
18	Yibin, Sichuan	mellow, a bit high-fired	group II
19	Yibin, Sichuan	sour, stale	group III
20	Lincang, Yunnan	mellow, sweet, brisk	group I
21	Lincang, Yunnan	sour, a bit smoky	group III
22	Yichang, Hubei	mellow, pure	group II
23	Yichang, Hubei	mellow, sweet	group I
24	Yichang, Hubei	mellow, sweet, brisk	group I
25	Yichang, Hubei	mellow, brisk	group II
26	Yichang, Hubei	mellow, brisk	group II
27	Yichang, Hubei	mellow, brisk	group II
28	Yichang, Hubei	mellow, a bit high-fired	group II
29	Yichang, Hubei	mellow, thick, a bit high-fired	group II
30	Yichang, Hubei	sour	group III
31	Yichang, Hubei	mellow, sweet, a bit high-fired	group I
32	Yichang, Hubei	mellow, brisk	group II
33	Yichang, Hubei	mellow, thick	group II

Table S2 Detailed information of the CAS number, supplier and purity of all the standards used in this study.

compound	CAS number	origin	purity
epigallocatechin, EGC	970-74-1	Yuanye bio-technology (Shanghai, China)	HPLC⩾98%
catechin, C	154-23-4	ZZBIO (Shanghai, China)	HPLC⩾95%
epigallocatechin gallate, EGCG	989-51-5	ZZBIO (Shanghai, China)	98%
epicatechin, EC	490-46-0	Yuanye bio-technology (Shanghai, China)	HPLC⩾98%
gallocatechin gallate, GCG	4233-96-9	ZZBIO (Shanghai, China)	98%
epicatechin gallate, ECG	1257-08-5	ZZBIO (Shanghai, China)	HPLC⩾98%
theaflavin, TF	4670-05-7	ZZBIO (Shanghai, China)	HPLC⩾98%
theaflavin-3-gallate, TF-3-G	30462-34-1	Yuanye bio-technology (Shanghai, China)	HPLC⩾98%
theaflavin-3′-gallate, TF-3′-G	28543-07-9	ZZBIO (Shanghai, China)	HPLC⩾98%
theaflavin-3,3′-digallate, TF-3,3′-DG	33377-72-9	ZZBIO (Shanghai, China)	HPLC⩾98%
phospho-L-serine	407-41-0	Sigma-Aldrich (St Louis, USA)	certified reference material grade
L-aspartic acid	56-84-8	Yuanye bio-technology (Shanghai, China)	HPLC⩾99%
L-threonine	72-19-5	Yuanye bio-technology (Shanghai, China)	HPLC⩾98%
L-serine	56-45-1	Yuanye bio-technology (Shanghai, China)	HPLC⩾98%
L-asparagine	70-47-3	Yuanye bio-technology (Shanghai, China)	HPLC⩾98%
L-glutamic acid	56-86-0	Yuanye bio-technology (Shanghai, China)	HPLC⩾99%
L-theanine	3081-61-6	Yuanye bio-technology (Shanghai, China)	HPLC⩾98%
L-proline	147-85-3	Yuanye bio-technology (Shanghai, China)	HPLC⩾99%
L-glycine	56-40-6	Yuanye bio-technology (Shanghai, China)	HPLC⩾99%
L-alanine	56-41-7	Yuanye bio-technology (Shanghai, China)	HPLC⩾98%
α-aminobutyric acid	2835-81-6	Yuanye bio-technology (Shanghai, China)	⩾98%
L-valine	72-18-4	Yuanye bio-technology (Shanghai, China)	HPLC⩾98%
L-cystine	56-89-3	Yuanye bio-technology (Shanghai, China)	HPLC⩾99%
L-methionine	63-68-3	Yuanye bio-technology (Shanghai, China)	HPLC⩾98%
L-isoleucine	73-32-5	Yuanye bio-technology (Shanghai, China)	HPLC⩾98%
L-leucine	61-90-5	Yuanye bio-technology (Shanghai, China)	HPLC⩾98%
L-tyrosine	60-18-4	Yuanye bio-technology (Shanghai, China)	HPLC⩾99%
γ-aminobutyric acid, GABA	56-12-2	Yuanye bio-technology (Shanghai, China)	HPLC⩾99%
L-histidine	71-00-1	Yuanye bio-technology (Shanghai, China)	HPLC⩾98%
L-tryptophan	73-22-3	Yuanye bio-technology (Shanghai, China)	HPLC⩾98%
L-ornithine	3184-13-2	Yuanye bio-technology (Shanghai, China)	HPLC⩾98%
L-lysine	56-87-1	Yuanye bio-technology (Shanghai, China)	HPLC⩾98%
L-arginine	74-79-3	Yuanye bio-technology (Shanghai, China)	HPLC⩾98%
vitexin-4”-*O*-glucoside	178468-00-3	Yuanye bio-technology (Shanghai, China)	HPLC⩾95%
myricetin 3-*O*-galactoside	15648-86-9	Yuanye bio-technology (Shanghai, China)	HPLC⩾98%
vitexin-2-*O*-rhamnoside	64820-99-1	Yuanye bio-technology (Shanghai, China)	HPLC⩾98%
quercetin-3-*O*-rutinoside, rutin	153-18-4	J&K scientific (Beijing, China)	98%
quercetin-3-*O*-glucoside	482-35-9	J&K scientific (Beijing, China)	98%
kaempferol-3-*O*-rutinoside	17650-84-9	J&K scientific (Beijing, China)	98%

kaempferol-3-*O*-glucoside	480-10-4	J&K scientific (Beijing, China)	98%
succinic acid	110-15-6	Yuanye bio-technology (Shanghai, China)	HPLC⩾98%
oxalic acid	144-62-7	Yuanye bio-technology (Shanghai, China)	HPLC⩾98%
malic acid	6915-15-7	Yuanye bio-technology (Shanghai, China)	HPLC⩾98%
citric acid	77-92-9	Yuanye bio-technology (Shanghai, China)	HPLC⩾98%
glucose	50-99-7	Jinsui biotechnology (Shanghai, China)	purity>99.8%
sucrose	57-50-1	J&K scientific (Beijing, China)	99%, analytical grade
gallic acid	149-91-7	J&K scientific (Beijing, China)	99%, analytical grade
caffeine	58-08-2	Sigma-Aldrich (St Louis, USA)	certified reference material grade

Detailed technical aspects of tea sensory evaluation

The sensory evaluation was performed in a clean, bright and quiet tasting room which is 15 m^2 large and is kept at 22 °C. All congou black samples were served in random order and coded with only regular numbers 1, 2, 3…, without further detailed information. All the panelists had no prior knowledge of these tested tea samples. For evaluation of each tea sample, a unified pack including a cup with a cover, a bowl and a spoon was used. All of them are white porcelain. The cup is cylindrical shaped and its size is height 65 mm, outside diameter 66 mm, inside diameter 62 mm, volume 150 mL. The bowl size is height 55 mm, volume 250 mL. At the mouth of the cup, there are three very small, zigzag shaped openings, for filtering of tea infusions into the bowl while keeping the leaves in the cup. Each tea sample was brewed in the cup with cover, then the tea infusion was filtered and transferred to the bowl for evaluation of tea taste. To make sure the reliability of the evaluation result, one tea sample was pre-selected from the experimental collection and brewed three times to make three replicates of tea infusions. These three infusions were set as blind samples and subjected to evaluation, along with other samples.

Analysis of free amino acids

The free amino acids were determined by high-performance cation-exchange chromatography with postcolumn derivatization, using an automatic amino acid analyzer (S-433D, Sykam, Germany) equipped with a cation separation LCAK07/Li column (4.6 mm×150 mm, Sykam). The mobile phase included buffer A (lithium citrate buffer: 5% (v/v) methanol with 5.04 g/L LiOH hydrate, 15 g/L citric acid, 0.01% (v/v) octanoic acid and 0.78% (v/v) of 37% HCL), buffer B (lithium citrate buffer: Water with 4.2 g/L LiCl, 8.4 g/L LiOH hydrate, 15 g/L citric acid, 0.01% (v/v) octanoic acid and 0.86% (v/v) of 37% HCL) and buffer C (lithium citrate/borate buffer: Water with 10 g/L borate, 4.2 g/L LiCl, 8.4 g/L LiOH hydrate, 10 g/L citric acid, 0.01% (v/v) octanoic acid and 0.33% (v/v) of 37% HCL). The flow rate was 0.45 mL/min and column temperature was 37°C. The injection volume was 10 μL. The LC gradient runs as following.

time/min	Buffer A/%	Buffer B/%	Buffer C/%
0.00	100.0	0.0	0.0
8.00	100.0	0.0	0.0
9.00	77.0	23.0	0.0
30.00	77.0	23.0	0.0
42.00	54.0	46.0	0.0
63.00	0.0	100.0	0.0
67.00	0.0	0.0	100.0
76.00	0.0	0.0	100.0
80.00	0.0	0.0	87.0
82.00	0.0	0.0	87.0
84.00	0.0	0.0	76.0
97.00	0.0	0.0	76.0
97.10	0.0	0.0	0.0
103.00	0.0	0.0	0.0
103.10	100.0	0.0	0.0
123.00	100.0	0.0	0.0
123.10	100.0	0.0	0.0
123.20	100.0	0.0	0.0
